# Keratinoctye-neuro-immune-units (KNICUs): collaborative impact on the initiation and maintenance of psoriasis

**DOI:** 10.3389/fmed.2023.1191057

**Published:** 2023-06-14

**Authors:** Xue-Yan Chen, Zhao-Yuan Wang, Yuan Zhou, Li-Ran Ye, Xiao-Yong Man

**Affiliations:** Department of Dermatology, Second Affiliated Hospital, Zhejiang University School of Medicine, Hangzhou, China

**Keywords:** psoriasis, epidermal immunological microenvironment, keratinocyte, immunocyte, neuron

## Abstract

The skin is the outermost barrier that separates the human body from the external environment. In psoriasis, immune cells reside within or infiltrate the epidermis to form the epidermal (epithelial) immunological microenvironment (EIME) and engage in complex interactions with keratinocytes, nerves, and microbiota. The proposed hypothesis is that psoriasis is a chronic inflammatory disease mainly mediated by a specific inflammatory environment composed of keratinocyte–neuro–immune cell units (KNICUs). These KNICUs arise from the interaction between activated epidermal keratinocytes, nerves, immune cells, and the skin microbiota, forming a complex interaction framework. Multiple units gather to complete the circulatory and amplified loops, consequently serving as a group army to initiate and maintain psoriasis.

## Highlights

- The immune and non-immune responses of the skin are composed largely of the EIME in the epidermis of psoriasis.
- Neuro-immune cell units (NICUs) have emerged as important structures in complicated processes such as inflammation in psoriasis.
- Keratinocytes are a trigger of inflammation in psoriasis.
- Psoriasis is mediated by a specific inflammatory environment composed of keratinocyte–neuro–immune cell units (KNICUs).

## 1. Introduction

The skin is the body's outermost barrier against environmental stressors, such as physical, chemical, and microbial agents. Within the skin, immune cells are present, encountering and responding to a myriad of inflammatory challenges (1). These cells are found in the epidermis, dermis, and subcutaneous fat. The epidermis comprises various cell types, such as keratinocytes, melanocytes, Langerhans cells (LCs), and other immune cells. Among those cells, LCs are considered the main skin-resident immune cells and antigen-presenting cells (APCs), located in the interfollicular epidermis and the epithelium of the hair follicles, while T cells, mainly CD8^+^ T cells, dendritic cells (DCs), and macrophages, are also involved in the network of APCs in recent research (2, 3). γδ T cells and CD8^+^ resident memory T cells (T_RMs_) are believed to be two specific T cells. CD8^+^ T_RM_ cells comprise non-circulating cells and have been identified to be caused by skin inflammation, which can be found in the stratum basal and stratum spinosum; these cells have also been discovered in the non-lymphoid organs, including the skin, where they can persist for an extended period of time (1). Immune cell populations in the skin predominantly consist of DCs and macrophages. However, the specific subtypes of DCs and their precise roles in the development of diseases are not yet fully understood. Subset-defining markers could be achieved by advances in single-cell separation technology.

Another evolving theory is that the skin immune responses are composed largely of the EIME in the epidermis of psoriasis. Epidermal cells not only secrete their cellular contents but also detect foreign and danger signals and then produce specific immune responses (4). Subsequent DC responses are also significant for inflammation (5). In addition, the epidermis and other factors, especially the microbiota and the peripheral nerves, contribute to the components of EIME in chronic inflammatory diseases (6).

Psoriasis is a chronic immunogenetic skin disease that affects ~1–3% of the population worldwide (7–9). However, the pathogenesis of psoriasis is still unknown. Historically, a prevailing theory indicates that excessive thickening of the epidermis in psoriasis is related to the abnormal proliferation of keratinocytes (10). Recently, neuroinflammation has been believed to be an important pathogenic element. Besides, the epidermis was considered the earliest stage of an inflammatory process (11), such as the pathogenic IL-17 loop. Keratinocytes are not only progenitors of the barrier but also participants in the innate immune system in the epidermis (1).

In contrast, the dermis is where immune cells and non-immune components interact (6, 12). However, recent research has identified the significance of keratinocytes as sensors of danger through the inflammasome systems in psoriasis (2). Furthermore, keratinocytes express several different pattern recognition receptors and produce a larger number of cytokines.

Neuro-immune cell units (NICUs) are anatomical units that exist at the interface of the immune and nervous systems, both in the states of health and when suffering from a disease (13). Clinical studies have identified that the peripheral nerves play a key role in the development of psoriatic lesions and that the injection of botulinum toxin type A is an effective treatment for psoriatic patients (14). In imiquimod-induced mice, skin inflammation was reduced by inhibiting the TRPV1^+^ sensory nerves (15). Besides, silencing sensory neuron-expressed transient receptor potential channel 4 (TRPC4) can induce psoriatic itch in the same mouse model (16).

Therefore, it is necessary to consider that the inflammatory reactions in the epidermis could be the center of immune responses and, together with NICUs, can be taken as one complete cycle to initiate the pathogenesis of psoriasis. The interaction of the epithelial tissue, immune system, and nerve systems completes each inflammatory loop, forming the huge circulatory inflammatory cycles in psoriasis.

## 2. Subsections

### 2.1. Epidermal (epithelial) immunological microenvironment

The skin is composed of the epidermis, dermis, and subcutaneous fat. It is presumed that the immune responses of the skin are determined and tightly associated with EIME in the epidermis and papillary dermis. There are two functional phases of EIME in the epidermis. First, keratinocytes are the body's barrier and can recognize dangers and external agents. Second, keratinocytes can amplify the immune reactions through EIME and produce cytokines that stimulate subsequent inflammation (6). Similarly, cytokines secreted by the immune cells can also affect the activation of non-immune cells, which together form an inflammatory network cycle.

Recent studies have reported three types of APCs present in the epidermis: LCs, inflammatory dendritic epidermal cells (IDECs), and monocyte-derived LC-like cells (17). IDECs and LC-like cells have been shown to be present in both steady and inflammatory states, but they increase more under inflammatory conditions such as atopic dermatitis and psoriasis (17). However, some studies have illustrated that LC is one type of dendritic epidermal cell, which includes four types. Among these, aLC/migLC (active LCs/migratory LCs) from the psoriatic skin showed higher expression of inflammation-associated molecules (18). Epidermal DCs are CCR2^+^ DCs, which are exclusively confined to psoriatic lesions and express different defining markers from dermal DCs (19). Other DCs found that CD1c^+^CD11b^+^ epidermal conventional type 2 DCs (cDC2s), CD141^+^ cDC1s, and BDCA2^+^ plasmacytoid DCs (pDCs) also played a significant role in psoriatic epidermis by using cyTOF (20). Thus, dendritic-like cells in psoriatic lesions are still inconsistent.

A review proposed that epithelial TRAF6, a type of ubiquitin E3 ligase, was associated with the immune responses and drove the inflammatory loops of IL-17 in the type 17 EIME in psoriasis, which also indicated the disordered homeostasis of epidermal NF-κB and MAPK signaling pathways. These were crucial for organizing the psoriatic EIME (4). Besides, neutrophil accumulation in the epidermis was the most important contributor to psoriatic EIME (6). The epidermal immune environment was more important and coincided with the inflammation occurring during psoriasis. EIME plays a dominant role in psoriasis, including psoriatic CD1c^+^CD11b^+^ epidermal cDC2s, CD141^+^ cDC1s, and BDCA2^+^pDCs (20). However, the specific role of immune cells and keratinocytes in psoriatic EIME is still not fully understood.

### 2.2. Immune cells in psoriatic skin

#### 2.2.1. DCs and epidermal DCs

A significant increase in DCs has been discovered both in the epidermis and the dermis in psoriatic lesions. DCs are typically classified into four groups: epidermal LCs, cDCs, plasmacytoid DCs (pDCs), and monocyte-derived DCs. Some types of DCs, such as TNF- and iNOS-producing DCs (Tip-DCs), slanDCs, and pDCs, are typically absent. However, in psoriatic skin, these DC types have been observed to primarily infiltrate the dermal component, but little is known about the specific classified types of DC in the epidermis of psoriasis (21). DCs have been believed to play a pivotal role in connecting innate immunity with adaptive immunity, which expresses TLRs and can sense danger signals from external agents. Besides, DCs are the source of IL-23 and TNF-a, which appear to have a central role in psoriasis pathogenesis (21). TLR stimulation can lead to the activation and maturation of DC, which are identified by the expression of costimulatory molecules and the secretion of cytokines (21).

Immune cell populations in the skin are dominated by DCs and macrophages (3). In an imiquimod-induced mouse model, in which MYD88 was only expressed by CD11c^+^ immune cells, the mice exhibited distinct psoriatic phenotypes compared to wild-type mice. These findings suggest that CD11c^+^ DCs are significant in psoriatic mice (22). Similarly, CD207^−^ DCs could produce IL-23 to stimulate dermal T cells to secrete IL-17 and IL-22 (23).

eDCs are CCR2^+^ DCs. They exceeded the number of LCs. The lesion would display an accumulation of neutrophils, the proliferation of keratinocytes, and the activation of T cells (19). These eDCs are capable of secreting IL-1β, IL-23, and TNF. Furthermore, research conducted by others has revealed the phenotypic and functional properties of eDCs and resident LCs in psoriasis. These cells play a role in amplifying the epidermal microenvironment through the secretion of IL-17 cytokines (24). eDCs have been observed to establish direct contact with hyperproliferative keratinocytes, a phenomenon that is strictly confined to active disease. Among the specific groups of eDCs involved, there may be cDC2s, CD141^+^ cDC1s, and BDCA2^+^pDCs (20). However, research on these eDC subsets in the context of psoriasis is still limited, emphasizing the need for further in-depth research in this area.

#### 2.2.2. Langerhans cells

A growing body of evidence has brought the identity of LCs into question, suggesting that they may originate from macrophage subsets rather than DC lineages, as previously presumed (17). LCs are currently reported as a specialized lineage of tissue-resident macrophages (25). However, the specific role that LCs play in the pathogenesis of psoriasis remains controversial (21, 23, 26–29). One study illustrated that LCs have an anti-inflammatory role during the process of psoriatic disease (21).

Other studies have reported that the migration of LC in non-lesional psoriatic skin is dependent on the IL-17 stimulation of keratinocytes. The inhibition of IL-17 has been found to restore normal LC migration. Besides, LCs have been shown to produce IL-23 and are required for the development of IMQ-induced psoriasis-like lesions (27). These studies, along with others, indicate that LCs may be truly involved in the pathogenesis of psoriasis (30, 31).

#### 2.2.3. Dendritic epidermal T cells

In the normal murine epidermis, the shape of several cell populations is included, including DETCs and epidermal lymphoid cells (ELCs) (32). DETCs are intraepithelial γδ T cells, which are identified by expressing Vγ5Vδ1 and have restrictive functions (33). The abilities of DETCs include inflammation modulation, cutaneous neoplasia protection, and skin wound healing (34, 35). The population of DETCs increases throughout adulthood until it reaches its peak, after which it remains stable (36). In the epidermis, DETCs could recognize a specific self-antigen that is restricted to damaged, stressed, or transformed keratinocytes (37). It is well-established that DETCs are potent IFN-γ-producing cells and that DETCs produce IL-17A upon TCR activation and in response to skin injury (38).

Human γδ T cells were only a small proportion of the total T cells in the dermis (2–9%) and the epidermis (1–10%), while in mice, DETCs account for more than 90% of epidermal T cells. DETCs are believed to negatively regulate αβ T cell-induced inflammation, thus contributing to local immune surveillance and immunoregulation, whereas αβ T cells promote skin tumor responses (39).

#### 2.2.4. Tissue-resident memory T cells

T_RMs_ are a novel non-circulatory T-cell subset that provides protective memory responses, maintaining long-term residence in the barrier tissues. Cutaneous CD8^+^ T_RMs_ reside in the epidermis and can last for approximately 1 year, whereas lung TRMs only last for 1 month. They are also considered to mediate autoimmune diseases such as vitiligo (40). The definition of T_RMs_ is the expression of CD69 in the absence of CD62L and the expression of T_RM_-associated molecules such as CXCR6 and CD103 or the transcription factor Hobit (41). The characterized marker of T_RMs_ is CD44^+^ CD62L^−^ CD69^+^. T_RMs_ can be distinguished from circulating lymphocytes by characteristically expressing CD69. Recent articles have reported that skin T_RMs_ in mice persist for more than 1 year. However, lung T_RMs_ last for only a few months (1). Although CD8^+^ T_RMs_ have been well-defined, CD4^+^ TRMs, especially skin-resident memory Th17 cells, are still being identified. T_RMs_ start a tissue-wide state of alert when recognizing their cognate antigen. They have the ability to recruit other immune cells to the site of infection (41).

In psoriasis, the skin affected by the condition displays an accumulation of IL-17-producing CD49a^−^ T_RMS_ (42). T_RMs_ derived from the skin are enhanced in the circulation of patients with PsA compared to patients with psoriasis alone (43).

### 2.3. Keratinocytes in psoriasis

The previous pathogenesis of psoriasis was presumed to, in accordance with inflammatory exudates in the dermis, be mainly composed of neutrophils, dendritic cells, T cells, and macrophages (44). The functions of the dermal immune cells have been widely explored, but the roles of epithelial cells have not been fully understood. Thus, we conclude that the two main roles of keratinocytes in psoriasis are as follows:

#### 2.3.1. Keratinocyte responses in psoriasis

The epidermis has been reported to be involved in determining the type of immune responses (6). Within the epidermis, keratinocytes, which are components of the innate immune system, express specific receptors and secrete cytokines. These cytokines include IL-6, IL-10, IL-18, IL-33, other IL-1 family members, thymic stromal lymphopoietin (TSLP), and tumor necrosis factor (TNF). Keratinocytes release these cytokines when the skin is inflamed (45). In addition, keratinocytes produce innate inflammatory mediators such as antimicrobial peptides (AMPs). AMPs have been found to be present at high levels in psoriatic lesions, which is related to the lack of skin infection in these patients. Moreover, keratinocytes can produce the cathelicidin antimicrobial peptide LL37 and thus break the self-tolerance mechanisms in psoriasis patients (2).

#### 2.3.2. Keratinocytes as a trigger of inflammation in psoriasis

Several studies have demonstrated that interlukin-17 (IL-17)-induced skin inflammation is targeted at keratinocytes (46). Th17 cytokines could activate keratinocytes in the inflammatory psoriasis loop and promote the release of more mediators such as CCL20. The production of neutrophil chemokines (CXCL1 and CXCL2) is one of the important roles of keratinocytes (47). In the pathogenesis of psoriasis, the DC responses might be stimulated by the abnormally dying keratinocytes in the stratum corneum. In addition, epidermal keratinocytes express several types of TLRs (48). TLR7 can be activated by stimulating TLR3 in keratinocytes, which is the most important signaling pathway in imiquimod-induced mice (49). Inhibiting the formation of isoleucyl-TRNA synthetase (IARS) in the epidermis may block the infiltration of immune cells in patients with psoriasis (50). A recent study by Moos et al. confirmed that the crosstalk between T cells, which produce IL-17, and keratinocytes facilitated the immune and non-immune responses that drove epidermal hyperplasia and cutaneous inflammation, which were critically dependent on keratinocytes. Since reduced inflammatory responses occurred only in mice with a specific deletion of IL-17RA in keratinocytes (10, 51), more and more evidence has shown that keratinocytes may trigger the progression of psoriasis (52). However, the specific role of other immune cells in psoriasis is still unknown.

### 2.4. Interactions between the nerve and immune system in the epidermis

Cutaneous innervation is critical in the peripheral nervous system (53). Epithelial neuro-immune cell units (NICUs) refer to specific anatomical locations. Neuronal and immune cells in NICUs can colocalize and functionally interact to regulate tissue pathology and physiology. NICUs have become an important structure for complicated processes such as inflammation, wound repair, and angiogenesis (13). In fact, in the epidermis, there is a colocalization of NICUs. Some studies have indicated that keratinocyte proliferation could result from cell–cell communication between keratinocytes and nerves by adding nerves to an HSE (54). Moreover, cutaneous nerve fibers interact with epidermal keratinocytes and immunocytes in the progression of psoriasis (55). Interactions between neurons and immune responses are mostly mediated by soluble factors such as neurotransmitters, neuropeptides, and cytokines due to keratinocytes having receptors such as neurotransmitters (e.g., acetylcholine, dopamine, adrenaline), neuropeptides, and neurotrophins, which are closely related to the psychoneuroimmunologic mechanisms (56).

### 2.5. Interactions between keratinocytes and nerve systemin in psoriasis

Cutaneous nerves consist of sympathetic and several sensory fibers, and the role of each fiber type remains unclear (15). The skin organ acts as both a sensory interaction place and a barrier. It is not only innervated by abundant sensory nerves but also by a smaller number of autonomic nerve fibers. Neurons form mesh-like bundles in the dermis, and there are abundant ends of nerves in the epidermis. A large fraction of dermal DCs' close contact with neurons in whole-mount immune staining indicate that neurons may regulate DC functions. To date, the functions of peripheral neurons in regulating immune responses in the skin have been unknown. However, through research involving the genetic and pharmacological ablation of TRPV1-expressing sensory neurons, it has become evident that neurons play a vital role in regulating cutaneous immune responses. This research has provided valuable insights into the regulatory role of neurons in the skin's immune system. For instance, after the pharmacological inhibition of TRPV1^+^ sensory nerves, significant suppression of mouse skin inflammation induced by the TLR7 ligand by imiquimod occurs (15). Following nerve deletion, IL-23-producing DCs in the dermis are reduced in imiquimod-induced inflammatory skin, and thus, the IL-17-releasing γδ T cells are clearly decreased (57). TRPV1^+^ sensory nociceptors directly recognize fungal agents in the *Candida albicans* infection model and then release calcitonin gene-related peptide (CGRP). Moreover, the production of IL-23 by CD301b^+^ DCs could be upregulated by CGRP, which in turn drives dermal γδ T cells to produce IL-17A (1). These results illustrated the possible interaction between keratinocytes and neurosystemin in psoriasis patients.

## 3. Discussion

**Figure 1:**
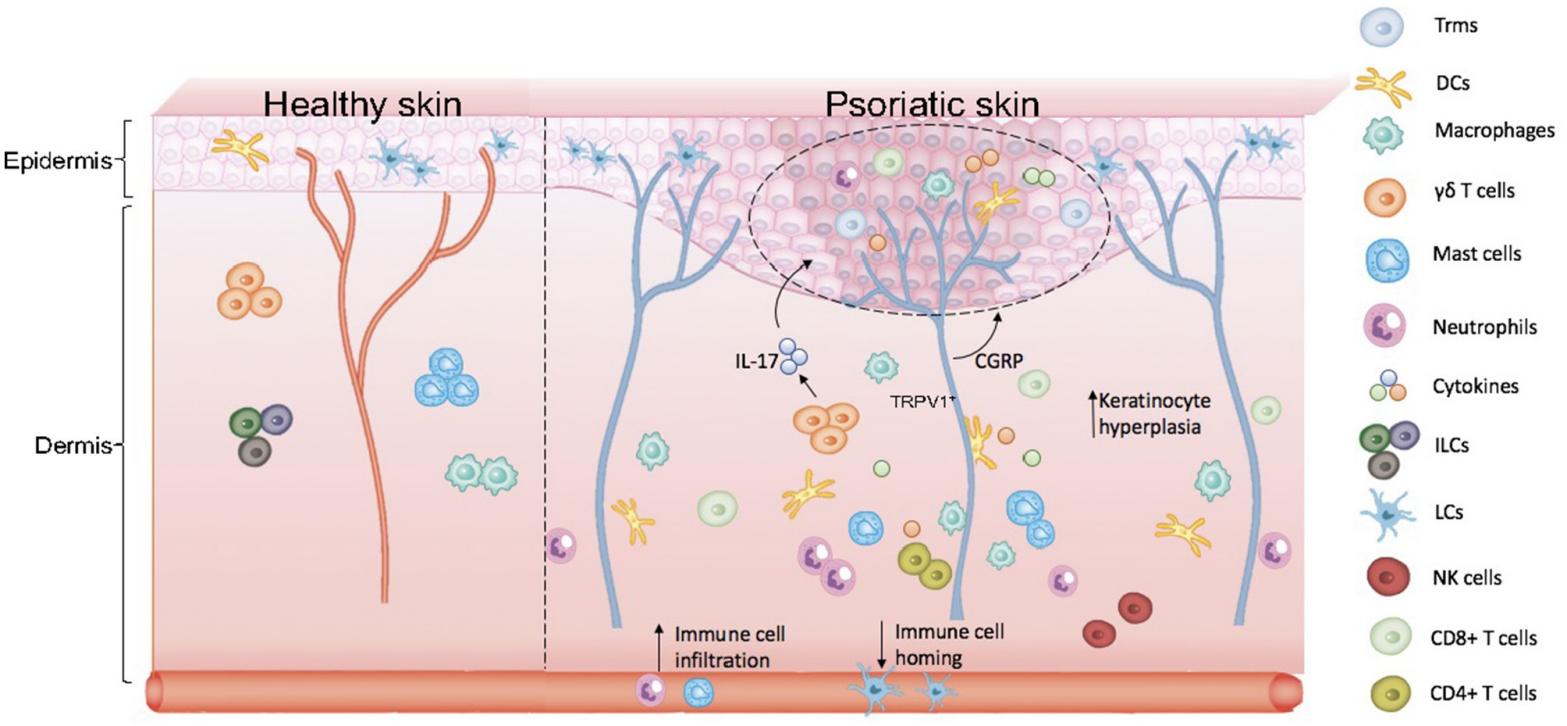
Skin anatomy and formation of keratinocyte–neuro–immune cell units, KNICUs. In psoriasis lesional skin loss, resident immune cells are largely activated. Dermal immune cells are immersed in the epidermis, with epidermal nerve fiber distribution increasing, which results in the abnormal proliferation of cells and the release of inflammatory factors. These cytokine factors, immune cells, and nerve fiber receptors—the three factors of the epidermis—form cell–neuro–immune cell units (keratinocyte–neuro–immune cell units, KNICUs). These constitute a complex feedback loop and cascade amplification effect, which plays a vital role in the development of psoriasis.

Based on the participation of EIME and NICUs in the pathogenesis of psoriasis, we proposed the concept of KNICUs in the epidermis, which could contribute to the establishment of an inflammatory environment where a large number of KNICUs interact with each other and subsequently perpetuate an inflammatory cycle in psoriasis. To substantiate this assumption, we need to fully understand the intrinsic mechanisms of KNICUs in all psoriasis types and phases, as shown in Figure 1.

In the past two decades, the pathogenesis of psoriasis has closely correlated with the functions of keratinocytes. Increasing evidence revealed that keratinocytes act as executors to actively participate in an immune-micro network interconnected by cytokines (56). The importance of keratinocytes in psoriasis leads to several new treatments and also raises new questions. For example, is it sufficient to target keratinocytes alone to induce psoriatic inflammation?

In the mouse model, TRPV1^+^ neuron activation in the skin was enough to initiate local type 17 immunity that augmented local host defense in mice, and type 17 innate immune responses achieved regional anticipatory immunity through the nerve (58). In addition, TRPV1 knockout mice treated with IMQ suggest a significant reduction in skin inflammation and barrier defects (59). The evidence showed that the interaction between nerves and immune cells is tightly associated with imiquimod-induced mice. However, the role of type 17 cytokines in the positive feedback loop of sensory nerves in psoriasis is still not fully understood.

We may obtain a deeper understanding of the mechanisms of cutaneous immune responses and of the pathogenesis of psoriasis by evaluating these points. Immune cells could express receptors for molecules derived from neuronal cells to respond to neuronal signals. Moreover, reciprocally, receptors for immune-derived cytokines and neurotransmitters are expressed by neurons. Hence, immune cells and neurons may interact with keratinocytes in psoriasis. Cytokine factors, immune cells, and nerve fiber receptors, the three factors of the epidermis, may form cell-neuro-immune cell units (keratinocyte-neuro-immune cell units, KNICUs). These constitute a complex feedback loop and cascade amplification effect, which are important to the development of psoriasis.

## Author contributions

X-YC and Z-YW: conceptualization and writing–original draft preparation. X-YM: funding acquisition. L-RY, YZ, and X-YM: writing–review and editing. All authors contributed to the article and approved the submitted version.
